# Critical Care Response to the Outbreak of COVID-19: The Experience From Guangdong Province, China

**DOI:** 10.3389/fpubh.2020.576528

**Published:** 2020-11-11

**Authors:** Xiaoqing Liu, Yonghao Xu, Zhiheng Xu, Yuanda Xu, Weiqun He, Yongbo Huang, Nanshan Zhong, Tiehe Qin, Yimin Li

**Affiliations:** ^1^State Key Laboratory of Respiratory Diseases, Department of Critical Care Medicine, Guangzhou Institute of Respiratory Health, First Affiliated Hospital of Guangzhou Medical University, Guangzhou, China; ^2^Guangdong Provincial Geriatrics Institute, Guangdong Provincial People's Hospital, Guangzhou, China

**Keywords:** ICU–intensive care unit, COVID-19, Guangdong Province, mortality, critical response

## Abstract

In December 2019, human infection with a novel coronavirus, known as SARS-CoV-2, was confirmed in Wuhan, China, and spread rapidly beyond Wuhan and around the world. By 7 May 2020, a total of 84,409 patients were infected in mainland China, with 4,643 deaths, according to a Chinese Center for Disease Control and Prevention report. Recent studies reported that critically ill patients were presented with high mortality. However, the clinical experiences of patients with coronavirus disease 2019 (COVID-19) have not been described in Guangdong Province, where by 7 May 2020, 1,589 people had been confirmed as having COVID-19 but with a very low mortality of 8 death (0.5%). Here, we describe the experience of critical care response to the outbreak of SARS-CoV-2 in Guangdong Province in the following points: Early intervention by the government, Establishment of a Multidisciplinary Working Group, Prompt intensive care interventions, Adequate ICU beds and Human resource in ICU, Infection control practices.

## Introduction

In December 2019, human infection with a novel coronavirus, known as SARS-CoV-2, was confirmed in Wuhan, China, and spread rapidly beyond Wuhan and around the world (1, 2). By May 7, 2020, a total of 84,409 patients were infected in mainland China, with 4,643 deaths, according to a Chinese Center for Disease Control and Prevention (CCDC) report (3). Recent studies reported that critically ill patients were presented with high mortality (4–8).

Sharing experiences of the management of coronavirus disease 2019 (COVID-19) patients from different centers are of vital importance for combating the pandemic. Fangcang shelter hospitals were rapidly built and responded to COVID-19 emergencies in Wuhan, China (9). Increasing intensive care unit (ICU) capacity and forecasting ICU demand were performed for more critically ill patients in the epicenter of Lombardy, Italy (10). However, the clinical experiences of patients with COVID-19 have not been well-described outside of the epidemic centers. Here, we introduce the critical care response to the outbreak of SARS-CoV-2 in Guangdong Province, where 1,589 people had been confirmed with COVID-19 but had a very low mortality of 8 deaths (0.5%) by May 7, 2020 (3).

## Early Intervention by the Government

Guangdong Province had initiated a level-one response to the public health emergency in January 2020. Under the coordination of the Government of Guangdong Province, the traffic and movement in the regions of Guangdong were restricted. Furthermore, early case detection, early reporting, early isolation, and contact quarantine, and early supportive care were conducted and led by the Government. Thus, the infected patients were effectively reduced, and the pandemic was effectively contained in Guangdong in a relatively short period.

## The Establishment of A Multidisciplinary Working Group

At the very beginning of the SARS-CoV-2 outbreak in Guangdong, a COVID-19 study group and an intensivist-led multidisciplinary team consisting of respiratory physicians, infectious disease specialists, and radiologists were established and participated in combatting the COVID-19 pandemic under the coordination of the Government of Guangdong Province. In addition, to help other cities in Guangdong, which faced challenges but had limited ICU resources, Guangdong released a “pairing-up support” plan. An expert team from each tertiary hospital in Guangzhou, the capital city of Guangdong Province, was paired up and sent to each designated hospital in remote areas across Guangdong. In a designated hospital, the intensivists made their rounds twice every day in the isolation wards and provided consultation for mild patients in these isolation wards. A rapid response team consisting of frontline health staff was also established for the care of patients with sudden clinical deterioration. All confirmed patients were screened for high-risk cases by the following measurements: (1) 5–7 days after onset; (2) age >50 years; (3) overweight patients, pregnant women, and children; (4) underlying health condition (e.g., diabetes, cardiovascular diseases, etc.); (5) persistent fever; (6) large lesion and/or more than two lesions in lungs; (7) fast decline in lymphocytes; (8) respiratory rate > 25/min and /or SpO2 <95% (during rest and breathing room air); (9) hypoxemia with normal heart rate; (10) altered mental status; (11) decreased appetite; and (12) dysfunction of extrapulmonary organs.

The above measures enabled the frontline health staff to identify patients with suddenly deteriorated statuses and intervene early to stabilize the patient. If patients presented with a respiratory rate >30/min, PaO_2_/FiO_2_ <250 mmHg (during rest and room air) or any signs of organ failure, they would be transferred to ICU for further monitoring and an intensivist-led multidisciplinary team would take over their treatment in a timely manner. In addition, the frontline health staff in the designated hospital would collect all new medical data of severe and critical cases every day and send them to the COVID-19 Data Center in Guangzhou. The data would be screened and discussed by the senior experts in the multidisciplinary team. A well-designed web-based video consultation system for critically ill patients with COVID-19 was established and applied across different cities in Guangdong Province. This allowed the multidisciplinary team to share their experience with the frontline health staff and help with the management of critical illness remotely across hospitals.

## Prompt Intensive Care Interventions

A research letter published in JAMA Network Open described 168 patients who died of COVID-19 from 21 hospitals in Wuhan which was the epicenter of the COVID-19 outbreak in China. The results showed that only approximately one-fifth of non-survivors with COVID-19 received invasive mechanical ventilation and further aggressive respiratory support prior to death, indicating that many patients had delayed intubation (11). The authors attributed the low proportion of invasive mechanical ventilation in non-survivors to the lack of invasive mechanical ventilators and patient management by a non-intensivist dominated medical team (11). Distinct from the characteristics of critically ill COVID-19 patients reported in Wuhan and other epicenters of the outbreak (4–7), patients in Guangdong were managed by a group of trained intensivists and specialist nurses with sufficient ventilators, extracorporeal membrane oxygenation (ECMO) equipment, and personal protective equipment. Moreover, as shown in our preprint data (12), the relatively low APACHE II and SOFA scores (the median APACHE II and SOFA scores was 14 and 4.0, respectively), of all patients at ICU admission indicated that fewer critically ill patients were admitted to the ICU to receive high intensity monitoring and prompt intensive care interventions. Besides, in our preprint study, 9 patients received ECMO due to a low but not too low PaO_2_/FiO_2_ ratio [85.2 (58.3–103.4) mmHg] or a high but not too high PaCO_2_ level [59.2 (53.5–68.9) mmHg], indicating early ECMO was initiated in patients presenting rapidly progressive respiratory failure (criteria for ECMO initiation: PaO_2_/FiO_2_ from 120 to 100 mmHg and/or PaCO_2_ from 50 to 55 mmHg, <4 h).

## Adequate ICU Beds and Human Resources in the ICU

The outbreak has led to a significant increase in the need for ICU beds. All designated hospitals in Guangdong actively prepared more ICU beds for the potential surge of critically ill patients with COVID-19. Meanwhile, the doctor/nurse-to-patient ratio was increased to intensively monitor the critically ill patients. Collectively, these measures may have contributed to the low mortality rate in Guangdong, which may reflect a lower mortality of the disease if there are adequate ICU beds, intensivists, and special nurses.

## Infection Control Practices

Strict isolation and protection measures were a top priority. Generally, confirmed patients were first isolated in single rooms with negative pressure. If more patients arrived, they were placed and treated in a closed unit. Medical staff were well-trained with handwashing, environmental cleaning, and had adequate personal protective equipment. Standard practices were established during airway management, including non-invasive mechanical ventilation (NIV), intubation, and bronchoscopy. According to the Chinese management guideline for COVID-19 (version 7.0), NIV can be considered for patients if standard oxygen therapy has failed (13). In our practices, for patients with NIV, they were monitored closely and cared for in a single room with negative pressure where intubation could be facilitated in the event of decompensation. In addition, an advanced, highly hydrophobic bacterial/viral filter (Iso-Gard HEPA Light, Item #: 28022) was connected to an NIV mask to reduce the production of aerosol generation. For intubation, an expert who specialized in the procedure was recommended. Endotracheal intubation was done in an airborne infection isolation room. The use of bronchoscopy was recommended for subglottic secretion drainage and tracheal aspirate in intubated patients. Personal protective equipment, including protective clothing, head coverings, double-gloving, N95 respirators, and eye protection, were provided during intubation and bronchoscopy. No medical staff were infected when dealing with patients with COVID-19 in Guangdong Province.

## Conclusion

The high mortality of patients in recent reports (4–8) may reflect the crisis of critical care medicine rather than the nature of COVID-19. Our experiences in Guangdong in the management of mild and critically ill patients with COVID-19 are of vital importance for intensive care colleagues in the community all around the world, who need to be prepared for any outbreaks in the future as it may became a seasonal threat for public health.

## Data Availability Statement

The raw data supporting the conclusions of this article will be made available by the authors, without undue reservation.

## Author Contributions

XL, YoX, ZX, NZ, TQ, and YL: conception and design. All authors: administrative support, provision of study materials or patients, collection and assembly of data, data analysis and interpretation, manuscript writing, and final approval of manuscript.

## Conflict of Interest

The authors declare that the research was conducted in the absence of any commercial or financial relationships that could be construed as a potential conflict of interest.
